# Internal structure, reliability and cross-cultural validity of the Warwick-Edinburgh Mental Wellbeing Scale in three European populations

**DOI:** 10.1136/bmjment-2024-301433

**Published:** 2025-03-11

**Authors:** Prateek Yadav, Jorge Arias de la Torre, Ioannis Bakolis, Xavier Bartoll, Cristina Casajuana Kogel, Joan Colom Farran, Alex Dregan, Carlos Garcia Forero, Lorena Botella-Juan, Vicente Martín, Antonio J Molina, Philippe Mortier, Line Nielsen, Katherine Perez, Beatriz Puertolas, Amy Ronaldson, Ziggi Santini, Ana Schiaffino, Antoni Serrano-Blanco, Sarah Stewart-Brown, Jose M Valderas, Jordi Alonso, Gemma Vilagut

**Affiliations:** 1 Institute of Psychiatry, Psychology & Neuroscience, King’s College London, London, UK; 2 South London and Maudsley NHS Foundation Trust, London, UK; 3 Care in Long Term Conditions Research Division, King’s College London, London, UK, UK; 4 CIBER Epidemiology and Public Health (CIBERESP), Madrid, Spain; 5 Institute of Biomedicine (IBIOMED), University of Leon, Leon, Spain; 6 Agència de Salut Pública de Barcelona (ASPB), Barcelona, Spain; 7 Institut de Recerca Sant Pau (IR SANT PAU), Barcelona, Spain; 8 Agència de Salut Pública de Catalunya (ASPCat), Barcelona, Spain; 9 Universitat Internacional de Catalunya, Barcelona, Spain; 10 Hospital del Mar Research Institute Barcelona, Barcelona, Spain; 11 University of Copenhagen, København, Denmark; 12 Capital Region of Denmark, Copenhagen, Denmark; 13 Institut Català d’Oncologia, l'Hospitalet de Llobregat, Llobregat, Spain; 14 Departament de Salut, Generalitat de Catalunya, Barcelona, Spain; 15 Institut de Recerca Sant Joan de Déu, Parc Sanitari Sant Joan de Déu, Barcelona, Spain; 16 University of Warwick, Coventry, UK; 17 Department of Medicine, National University of Singapore, Singapore; 18 Department of Family Medicine, National University Health System, Singapore; 19 Centre for Research in Health Systems Performance (CRiHSP) National University Health System, Singapore; 20 Department of Experimental and Health Sciences, Universitat Pompeu Fabra, Barcelona, Spain

**Keywords:** Data Interpretation, Statistical, Cross-Sectional Studies

## Abstract

**Background:**

The Warwick-Edinburgh Mental Wellbeing Scale (WEMWBS), a questionnaire designed for the assessment of mental well-being, is widely used in different countries and cultures worldwide. However, there is a lack of studies examining its metric performance and measurement invariance across countries.

**Objective:**

This study aims to examine the internal structure, reliability and cross-country validity of the WEMWBS in three European populations.

**Methods:**

WEMWBS data collected in 2016 from three representative population health surveys from an autonomous region in Spain (Catalonia) and two countries (Denmark and the UK) were used (n=13 940). The mean WEMWBS Scores were compared between populations. The internal consistency (ω coefficients), internal structure (confirmatory factor analyses (CFA) and bifactor exploratory structural equation models), reliability (item response theory models, item and test information functions), and cross-cultural comparability (multigroup CFA) of the WEMWBS were assessed.

**Findings:**

Differences in mean scores observed between regions merit further study. The WEMWBS showed high internal consistency across countries (ω=0.942). The unidimensionality of the scale was confirmed overall and for each population. Evidence of reliability and of measurement invariance at the configural, scalar and metric levels was found.

**Conclusions and implications:**

The results support the use of the WEMWBS in different cultures to inform the understanding of population well-being in public health and its possible use as an outcome measure in clinical studies.

**WHAT IS ALREADY KNOWN ON THIS TOPIC**

- Mental well-being is arousing great interest as an outcome measure from a public health and clinical perspective, worldwide.
- One of the most widely used tools specifically designed for the assessment of mental well-being is the Warwick-Edinburgh Mental Wellbeing Scale (WEMWBS).
- However, evidence about its internal structure, that is, if it measures just mental well-being or also other constructs, and reliability is still sparse, and the comparability of the WEMWBS Scores between different cultural contexts remains unclear.

**WHAT THIS STUDY ADDS**

- The results from this study, one of the largest to date, specifically focused on the assessment of mental well-being and based on large and representative samples from three different cultural contexts in Europe (the UK, Denmark and Catalonia), provide additional robust evidence of the primary focus of the WEMWBS on general mental well-being and advocate for its application as a unidimensional measure.
- Also, the results reinforce the reliability and validity of the WEMWBS as a tool for assessing mental well-being in these contexts and demonstrate its measurement invariance across them.

**HOW THIS STUDY MIGHT AFFECT RESEARCH, PRACTICE OR POLICY**

- As far as we know, this study is the largest cross-cultural validation of the WEMWBS, confirming the cross-cultural validity of the WEMWBS across diverse European countries, supporting the suitability of the scale as a relevant unidimensional outcome measure for the assessment of mental well-being at the population level, for its use in multicultural population studies, clinical settings and in other contexts, at least at the European level.
- The availability of a measure with these characteristics allows us to determine and compare the levels of mental well-being in different contexts, determine the effectiveness of public health and clinical interventions to improve it and, consequently, to increase it at the population level.

## Introduction

 Mental well-being, also known as positive mental health, is a key priority of the WHO and the Organization for Economic Cooperation and Development recommendations,^1 2^ and is gaining increasing relevance from a public health and clinical perspective, worldwide. Despite its increasing relevance, measuring mental well-being is complex because mental well-being is not simply a lack of mental health, but a multifactorial combination of many factors, and both hedonic and eudaimonic perspectives must be considered.

In the absence of reliable detectable biomarkers or investigations for the assessment of mental well-being, self-reported questionnaires are an important resource for its measurement as they are able to provide quantitative data on experiences, feelings and functioning.^3^ One of these questionnaires, likely the most widely used, is the Warwick-Edinburgh Mental Wellbeing Scale (WEMWBS), a self-report questionnaire to measure mental well-being, encompassing both hedonic well-being (feelings of happiness and satisfaction) and eudaimonic well-being (ie, functioning, purpose in life, personal development and meaningful relationships).^4^ It consists of 14 Likert-type items describing components of mental well-being phrased as positive statements, as opposed to components of mental ill health phrased in the negative.^4^ While higher WEMWBS Scores are associated with lower scores for psychopathology, mental well-being goes beyond the mere absence of mental illness, and it correlates with measures of health-related behaviours and physical capability.^4^ This is a reason why the WEMWBS has been used as an outcome measure in clinical studies and in a wide variety of non-clinical settings, including the commercial and third sectors and education systems. Besides, a previous systematic review and meta-analysis assessing interventions to improve well-being using changes in WEMWBS Score as the main outcome measure found that the 14-item WEMWBS version could be considered, due to its flexibility, a relevant outcome measure in a wide range of medical, public health and social interventions.^5^ This points out the WEMWBS value as an outcome measure in health services settings, but also at the population level to inform the development of public health policies.

Even though the WEMWBS was initially developed as a unidimensional scale,^6^ the factor structure of the measure has been subject to substantial debate, and alternative structures have been explored. Some studies have proposed two-factor models, including hedonic and eudaimonic well-being,^7^ or a three-factor structure including an additional separate social factor.^8^ Recent studies exploring the dimensionality of the WEMWBS have added to the complexity, with a bifactor structure with a general mental well-being factor alongside specific subfactors on hedonia, eudaimonia or personal relationships demonstrating superior fit than one-factor to three-factor models.^8 9^ Bifactor models are particularly advantageous, as they simultaneously account for the general construct and specific variances,^10^ and offer a comprehensive and methodologically robust approach to evaluating the dimensionality of the WEMWBS. However, further research is needed to determine whether they offer meaningful improvements in interpreting WEMWBS Scores over simpler structures.

The WEMWBS was originally developed and validated in the UK but has been translated and adapted for use in many other populations across the world. Although the WEMWBS has been extensively validated for its use in individual countries, including France, the UK, Denmark and Spain,^4 11 12^ to date there is a lack of evidence about its cross-cultural comparability. One of the largest direct cross-country comparisons to date in Europe involved data collected in Denmark, Iceland, Catalonia and the UK.^13^ However, it addressed WEMWBS validity in Denmark only and did not assess cross-cultural validity between the countries.^13^ Besides, a comparison of WEMWBS between Catalonia and Scotland has been performed, which showed significantly different mean WEMWBS Scores between these countries.^14^ Differences in social and cultural contexts may influence how individuals interpret and respond to items, potentially altering the scale’s dimensionality. It is essential to ensure that measures of pathology and mental well-being are both reliable and cross-culturally valid—that is, equivalent and comparable across diverse cultural and population groups. A lack of comparability can result in misinterpretations of results, thereby diminishing their utility as measures for informing evidence-based decision-making in mental health. Given the limited research on the cross-cultural validity of the WEMWBS, conducting studies using data from several populations is instrumental in determining its relevance as a mental well-being measure.

In this context, this study aims to investigate the internal structure, reliability and cross-cultural validity of the WEMWBS in a secondary data analysis of population health survey data from three European populations.

## Methods

### Participants and data

A cross-sectional study based on representative data from population health surveys from Catalonia (Spain), Denmark and England was carried out. Data from Catalonia (Spain) were obtained from the 2016 Catalan Health Interview Survey (ESCA).^15^ Anonymised data from ESCA were requested and accessed after signing a data transfer and confidentiality agreement with the Department of Health of the Government of Catalonia. Catalonia is a region of Spain with a population of 7.7 million in 2022. Respondents were non-institutionalised persons in the general population aged 15 years and over selected from the Population Registry of Catalonia of the Catalan Institute of Statistics. The sample was selected through a stratified three-stage random sampling strategy. The computer-assisted interviews were conducted face to face by trained interviewers at the respondents’ residences. Data from the two waves of the survey carried out in 2016 were used, with a total sample size of 4818, but only data on individuals 16 years and over with complete WEMWBS data were extracted and used (n=3651).^15^

The data from Denmark comes from the Danish Mental Health and Wellbeing Survey 2016 (n=3508).^16^ This survey used a random representative sample of Danish individuals from the Danish Civil Registration System 16 years and older.^17^ Denmark had a population of 4718 756 at the time of the survey. The survey and sampling design were carried out by Statistics Denmark. Individuals were randomly selected and invited by a letter via digital mail, with information about the survey and instructions for the online questionnaire. Two reminder letters (one postal and one digital) were sent to non-responders. A total of 10 250 individuals were sampled, of which 3508 participated in the survey (34.2%). From these participants, 174 were excluded due to missing data in all or some of the WEMWBS items (4.96%).

Data from the UK were taken from the 2016 National Health Survey for England carried out by the Joint Health Surveys Unit of NatCen Social Research and the Research Department of Epidemiology and Public Health at University College London. Data were requested and accessed through the UK data service, and use of safeguarded data is governed by a legally binding end user licence agreement, which forms part of the registration process. The survey involved a stratified multistage, random probability sample of the non-institutionalised population living in private households in England.^18^ Addresses were randomly selected from 531 postcodes to provide a representative sample. The survey was carried out using face-to-face interviews and a self-reported module to be completed by the participant, including the WEMWBS Questionnaire. A total of 8011 individuals 16 years or older participated (response rate=55%), of which 7153 (89.3%) completed the self-reported module. 198 of these participants were excluded due to missing data of all or some of the WEMWBS items (2.8%).

All data collected from the population surveys were anonymised. Confidentiality and privacy requirements were met through complete anonymisation and through users’ licence agreements with the corresponding organisations whenever required. Only data from those over 16 years with complete WEMWBS responses were included to ensure comparability between countries (n=13 940).

### Outcome measure

The WEMWBS is a self-report questionnaire consisting of 14 Likert-type items about the last 2 weeks, with a 5-point response scale assessing eudaimonic (eg, people’s functioning, social relationships, sense of purpose) and hedonic (eg, feelings of happiness) components of mental well-being. A higher score corresponds to a higher level of mental well-being, with scores ranging from 14 to 70.^4^ The WEMWBS shows suitable psychometric properties for the assessment of mental well-being for each of the populations included in this study (the UK, Denmark and Spain).^411^,^13^

### Statistical analyses

#### Descriptive analyses

Descriptive statistics were calculated including the mean, median, IQR and SD. Kruskal-Wallis tests were carried out to assess possible differences between countries due to the non-normal distribution of the WEMWBS Scores.

#### Internal structure and internal consistency

To assess the internal structure of the questionnaire, we compared several factor structures based on previous research: (A) A one-factor confirmatory factor analyses for categorical items (iCFA) model;^6^ (B) A two-factor iCFA model, including hedonic and eudaimonic dimensions;^7^ (C) A three-factor iCFA model with hedonic, eudaimonic and social relations factors;^8^ (D) A three-factor model with a slightly different distribution of items within factors;^19^ (E) A second-order three-factor iCFA based on the previous best-fitting three-factor structure;^19^ (F) A bifactor exploratory structural equation model (bifactor ESEM) with target rotation with a general factor and three specific factors.^8^ All models were estimated for each population and for the whole data set. The weighted least squares estimator with robust adjustment for mean and variance was used.^20^ To assess and compare the goodness of fit of the different models, the following statistics were calculated: the χ^2^, Comparative Fit Index (CFI >0.95 for good fit), Tucker-Lewis Index (TLI >0.95 for good fit), root mean square error of approximation (RMSEA <0.06 for good fit, <0.08 for acceptable fit) and standardised root mean square residual (SRMR <0.08 for good fit).^21^ Based on the bifactor ESEM model, several psychometric indices were obtained that collectively help determine whether subscale scores provide unique information above and beyond the total score, and to what extent the total score can be interpreted as a reliable and valid measure of the intended construct, despite the presence of multidimensionality.^22^ The indicators studied were: the explained common variance (ECV), or the proportion of all common variances explained by each factor. Values of ECV ≥0.85 suggest that the scale is sufficiently unidimensional. The ω reliability coefficient for the multidimensional composite total score (ie, the proportion of the total score variance that can be attributed to all common variances), the ω hierarchy for each factor (*ωH*) (that is, the proportion of total score variance that can be attributed to each common factor), indicates the degree to which the raw score reflects the target dimension, and the average relative parameter bias (ARPB), that is, relative difference between item loadings from the unidimensional solution and those from the general factor in the bifactor, that is, the truer model. Additionally, Cronbach’s α and McDonald’s ω were calculated to assess the internal consistency across the items within the WEMWBS.

#### Item response theory

For an item response theory (IRT) approach, the following weighted polytomous IRT models were fitted: the partial credit model (PCM), the generalised partial credit model (GPCM) and the graded response model (GRM). The model that best fitted the data was selected using the Akaike Information Criterion. Additionally, the item information functions to evaluate the amount of information provided by each item at a given ability level were plotted for each item across countries.^23^ Also, test information functions to assess information of the whole test score throughout the latent continuum (θ_i_) were plotted for each country and the whole data set.^23^ The higher the information value, the greater the precision of the measurement. Score reliability throughout the continuum was estimated as 1 − (1/Information (θi)).

#### Measurement invariance

To assess the correspondence of the WEMWBS across countries, multigroup CFA were carried out to assess the configural (ie, the consistency of the latent structure of the scale across all groups), metric (ie, whether the items are related to the latent trait of the scale in an equivalent way in all groups) and scalar invariances (ie, whether the items show the same expected response across all groups).^24^ Weighted least squares estimator with robust adjustment for mean and variance was used. To assess the presence of measurement invariance, the following goodness-of-fit statistics were calculated for a one-factor model: χ^2^, CFI, TLI, RMSEA and SRMR.^21^ Comparisons were made between the different invariance levels by calculating the absolute differences in the fit statistics across levels as proposed by Chen.^25^ Invariance models are considered to provide an adequate fit if changes from one level of invariance to another in CFI and TLI remain below 0.010, below 0.015 in RMSEA and below 0.030 in SRMR.^25^

All analyses were weighted using the weights derived from the complex sampling strategies for each of the surveys. All analyses were carried out using the Stata V.17 MP and Mplus 8 software packages.

## Results

### Descriptive statistics

The demographic characteristics for the data from each region and for the total sample can be found in online supplemental appendix A. The mean WEMWBS Score was highest in Catalonia (M=58.6, 95% CI 58.3 to 58.8), followed by Denmark (M=52.2, 95% CI 51.9 to 52.5) and the UK (M=49.7, 95% CI 49.5 to 50.0). The differences between countries were statistically significant (p<0.001) (table 1). Additionally, a forest plot showing mean WEMWBS Scores (and 95% CI) for each country and for the total sample can be found in online supplemental appendix B.

**Table 1:** Descriptive statistics for the Warwick-Edinburgh Mental Wellbeing Scale (WEMWBS) by country

Country	N	Mean	SD	95% CI	Median	IQR
Catalonia (Spain)	3651	58.6	8.3	58.3 to 58.8	59	55–65
Denmark	3334	52.2	8.7	51.9 to 52.5	53	47–58
The UK	6955	49.7	8.8	49.5 to 50.0	50	44–56
Total	**13 940**	**52.7**	**9.4**	**52.5 to52.8**	**54**	**47–59**
Kruskal-Wallis *value of p*	<0.001					

### Internal structure and internal consistency

The goodness-of-fit statistics for the different models estimated are shown in online supplemental appendix C, for the total sample and each region. As expected, given the large sample size, the χ^2^ statistic was significant for all the models. Results for the unidimensional model initially hypothesised provide adequate CFI and the TLI values above 0.90 for the overall and for each of the three countries included, and good SRMR lower than 0.05. However, the RMSEA values around 0.10 or higher for all populations indicate inadequate fit for the one-factor model according to this indicator. The bifactor ESEM model with target rotation provided the best fit to the data for all populations, with consistently all goodness-of-fit indices over the prespecified cut-off points indicating good (CFI=0.990, TLI=0.977, SRM=0.014 values for the total sample) or adequate (RMSEA=0.075, 90% CI 0.073 to 0.077 in the total sample) fit.

The full standardised factor loadings from the bifactor ESEM model, which provided the best fit to the data, can be found in the table in online supplemental appendix D. Item 8 (*I've beenfeeling good about myself’*) had the greatest standardised factor loading on the general factor, indicating the highest correlation between the item and well-being, in the data from Denmark and from the UK, and the total sample. In Catalonia, item 10 (‘*I've been feeling confident’*) had the greatest loading in the general factor. Loadings for specific factors were generally small to moderate, with values below 0.4 for most items.

Several psychometric indicators were obtained from the bifactor model (online supplemental appendix E), showing very consistent results across populations. ECV for the general factor was 0.86 for the total sample (ranging from 0.82 in UK and Denmark and 0.85 in Catalonia) and ω H values for the general factor over 0.92 for all populations. ARPB values in all populations were around 4%, well below the 10%–15% cut-off indicating low bias introduced when assuming unidimensionality. All these indicators consistently support the predominantly unidimensional structure of the scale across the three countries and justify the interpretation of the total raw score.

The internal consistency estimates Cronbach’s α and McDonald’s ω coefficients (based on the unidimensional model) for the WEMWBS in the overall sample and in each population are provided in table 2. Both α and ω values were over their respective thresholds (*α>0.85, ω>0.70*) for each population and total, indicating high internal consistency and reliability of the WEMWBS across populations.

**Table 2:** Cronbach’s α coefficient and ω coefficient of the Warwick-Edinburgh Mental Wellbeing Scale (WEMWBS) by country

Country	N	Cronbach’s α coefficient*	ω coefficient†
Catalonia	3651	0.934	0.953
Denmark	3334	0.921	0.941
The UK	6955	0.931	0.959
Total	**13 940**	**0.923**	**0.942**

* α must be greater than 0.85 for the scale to be considered reliable (calculated using standardised factor loadings from a weighted factor analysis for categorical items).

† ω based on the unidimensional model must be greater than 0.70 for the scale to be considered reliable (calculated using the weighted variance of each item and of the total items).

### Item response theory

The GRM had the best fit of the different weighted polytomous IRT models, showing the lowest Akaike information criterion, compared with the PCM (total=40.08) and the GPCM (total=39.38). The results of the Akaike information criterion for the different models and for the different populations are provided in online supplemental appendix F. The results from the GRM show that item 8 (‘*I've been feeling good about myself’*) is the item with the greatest ability to discriminate mental well-being in the UK and in Denmark. They also show that in Catalonia item 10 (‘*I've been feeling confident’*) is the one with the greatest discriminating capacity. The item-specific discrimination parameters for the GRM are shown in online supplemental appendix G.

**Figure 1:**
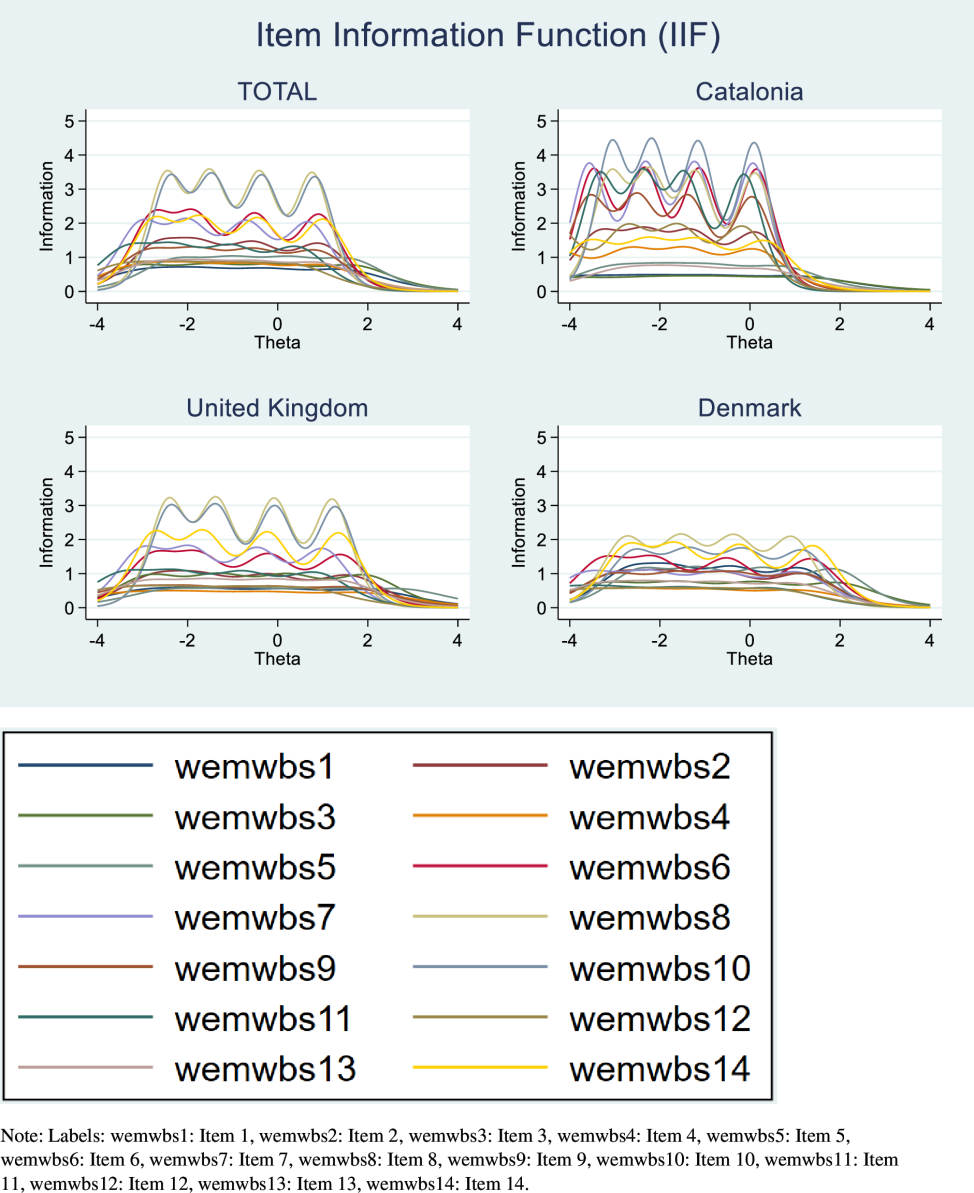
Total item information function (IIF) for each item and for each item by country. WEMWBS, Warwick-Edinburgh Mental Wellbeing Scale.

The item information functions are shown in figure 1. In the total sample, items 10 and 8 had the greatest reliability. In Catalonia, item 10 (*‘I’ve been feeling confident’*) can be seen to have the greatest reliability. In the UK, both items 10 and 8 (‘*I’ve been feeling good about myself’*) appear to have the greatest reliability. Data from Denmark do not show any item that clearly stands out from the others. However, item 8 appears to have slightly higher reliability compared with other items. Online supplemental appendix H shows the test information function for all countries together and for each country. In all countries, a reliability ≥0.90 was observed in the negative part of the continuum of the latent trait. Data from Catalonia had the highest reliability, followed by Denmark, and then the UK.

### Measurement invariance

Table 3 shows the fit statistics for configural, metric and scalar invariance for the WEMWBS based on the unidimensional model. All fit statistics for the configural invariance model indicated an adequate fit, except for the RMSEA, which was >0.08, indicating inadequate fit. This shows that the underlying latent structure is found to be consistent across the three countries, in four out of five of the tests of fit. Also, the metric invariance model, where restrictions were added to test the equality of factor loadings between countries, showed a good fit for all tests with the exception of the RMSEA. Besides, the comparison between the configural and metric invariance models, using differences in the fit statistics CFI, RMSEA and SRMR, also confirmed that the latent structure is consistent across countries (ΔCFI=−0.006, ΔTLI=−0.013, ΔRMSEA=0.013, ΔSRMR=0.000). Only the difference in the TLI adjustment statistic showed a difference slightly larger than the threshold (−0.013, when the difference should be smaller than 0.010). Finally, when constraints were added to test for equality of thresholds*,* that is scalar invariance, all model fit statistics indicated a good fit except for the RMSEA, and when comparing the metric invariance model with the scalar invariance model, the differences in all the fit statistics met the criteria proposed by Chen,^25^ confirming good fit (ΔCFI=−0.004, ΔTLI=0.007, ΔRMSEA=0.008, ΔSRMR=−0.004).

**Table 3:** Configural, metric and scalar invariance for the items of the Warwick-Edinburgh Mental Wellbeing Scale (WEMWBS)

Invariance	^χ2^	CFI	TLI	RMSEA	SRMR
Configural	13 807 087	0.953	0.944	0.112 (0.111 to 0.114)	0.041
Metric	11 981 545	0.959	0.957	0.099 (0.098 to 0.101)	0.041
Scalar	13 281 981	0.955	0.964	0.091 (0.089 to 0.092)	0.045

Note : Ffit statistics : χ2: Chi-Square test;

χꭓ2 with 231 *degrees of freedom* and *p*p<0.001 for the configural invariance model

χꭓ2 with 257 *degrees of freedom* and *p*p<0.001 for the metric invariance model

ꭓχ2 with 339 *degrees of freedom* and *p*p<0.001 for the scalar invariance model

CFI, Comparative Fit Index ; RMSEA, root mean square error of approximation ; SRMR, standardised root mean square residualTLI, Tucker-Lewis Index

## Discussion

This study is, as far as we know, the first study to assess over three culturally different populations. The internal structure, reliability and cross-cultural validity of a tool specifically designed for the assessment of mental well-being, the WEMWBS were assessed. Despite the similarities between the populations (all from Europe), and although there were differences in mean well-being scores across the populations assessed, the WEMWBS showed adequate internal consistency, reliability and cross-country comparability. These findings validate the 14-item version of the WEMWBS as a robust unidimensional measure of mental well-being across diverse settings and cultures, reinforcing its suitability for international use, showing measurement invariance at the European level. These results provide valuable support for the measurement of well-being and inform the development of international public policies.

Looking at previous studies focused on the WEMWBS, it should be noted that they have been smaller in scope than the present study. A cross-cultural evaluation of the WEMWBS in Chinese and Pakistani origin populations living in the UK found high levels of consistency and reliability; however, this was only in one country.^26^ Additionally, in line with the results found, a previous study comparing data from Catalonia and Scotland found significantly different WEMWBS Scores and also demonstrated reliability of the WEMWBS in both countries.^14^ Besides, to our knowledge, the largest direct cross-country comparison to date of the WEMWBS involved data from Denmark, Iceland, Catalonia and the UK, but it only assessed validity in Denmark.^13^ Thus, while larger studies involving other populations from different countries worldwide are needed, the results found show the potential of the WEMWBS as a mental well-being measure to be used in multicultural studies and contexts.

The difference in mean WEMWBS Scores between countries is consistent with previous research comparing them.^13^ On the other hand, these findings are inconsistent with results from international assessments of happiness and well-being. The World Happiness Report (WHR) and the European Social Survey (ESS) both rated Denmark higher for happiness and well-being than the other regions in this study.^27 28^ This may be due to differences in constructs used for the assessment of well-being. The ESS uses a wide array of questions over many life domains and the WHR used a Cantril Ladder Scale alongside topics as varied as measures of state effectiveness, freedom and social support. A Cantril Ladder involves participants rating these aspects of their life on a scale of 1 to 10. As a result, the ESS and WHR may give a broader view of well-being alongside many other factors (eg, socioeconomic development, political system, environmental factors) and not specifically mental well-being, leading to the difference in results. The WEMWBS provides a more focused measure of mental well-being. To stand out as a tool that can be used both as a population measure and clinical outcome measure needs concise and narrower focus. Furthermore, the conciseness of the WEMWBS lends itself well for use in large-scale population surveys and studies.

The results on the internal structure of the WEMWBS support a strong general well-being factor, consistent with prior studies examining the scale’s dimensionality using bifactor models.^8 9 19^ The high values obtained for ECV and the ωH coefficient and low ARPB underscore the unidimensional interpretability of the scale for mental well-being. As far as we know, only one other study has calculated some of these indicators, obtaining consistent results.^19^ The results also show high reliability of the WEMWBS, with minor differences in which items were most discriminatory in each country, which were consistent over different tests. Potential similarities and differences in language, culture and cultural understanding of well-being might lead to differences in items performing between the different countries. This is a well-described phenomenon for self-reported questionnaires using Likert-type items, as the possible differences in response styles over different countries, and the language and translation used, could affect the responses given.^29^ Factors affecting responses appear to include country-level features such as extraversion, collectivism and uncertainty avoidance influencing response styles.^29^ However, these factors were not considered in the surveys included in the study and, hence, further research is required to assess the role of these factors in relation to the WEMWBS performance in different cultures.

Several limitations of the study need to be discussed. First, it only assessed data over three populations from Europe, and this could be expanded to include more populations from other regions worldwide in which mental well-being would be considered conceptually different, such as India or African nations. Second, the cross-sectional nature of the data used for this study and the lack of follow-up of participants preclude testing the WEMWBS validity from a longitudinal perspective. Furthermore, the sample from Catalonia is only from one region of a country, while the other two samples are countrywide samples (Denmark and the UK). Differences in mean scores between different regions could be explored further and could be due to a variety of factors in the three regions. Further analysis in future work adopting a longitudinal perspective could be done regarding the validity of WEMWBS and the differences in its scores between demographic groups, for example, gender and age group. Another potential avenue is exploring the reasons for the similarity of discriminatory capacity and reliability across many items in the WEMWBS, which may indicate redundancy. Indeed, the original authors of the WEMWBS developed a Short Warwick Edinburgh Mental Wellbeing Scale (SWEMWBS) including only seven items.^30^ However, it should be noted that they acknowledge that the SWEMWBS may present a narrower and more restricted view of well-being than the original WEMWBS and the SWEMWBS questions skew more towards eudaimonic and psychological well-being with little coverage of hedonic well-being.^30^ Additionally, it must be highlighted that the scope of this study is the assessment of the cross-cultural validity of the original and complete WEMWBS, and not the development of a shorter version of the questionnaire. Further research to develop a shorter and cross-culturally valid version of the WEMWBS could be helpful to disentangle these aspects.

To our knowledge, this is the first cross-cultural validation study of the WEMWBS, comparing three different European countries, adding evidence supporting its use and comparability. The results found highlight its potential to be used as a measure of mental well-being in Europe and to make comparisons of mental well-being between countries. Furthermore, it supports the potential of the WEMWBS to be used as an outcome measure in multicultural and multicountry clinical and epidemiological studies in Europe. Despite the fact that further work is needed to assess the cross-cultural validity of the WEMWBS in other European countries and outside of Europe, the results show the validity of using the WEMWBS in surveys and studies across multiple cultures, aiding assessment and potential improvement of population well-being at the population level.

## supplementary material

10.1136/bmjment-2024-301433 online supplemental file 1

## Data Availability

Data sharing is not applicable as no data sets were generated and/or analysed for this study. Data are available upon reasonable request.
