# Supplementary material for: Internal structure, reliability and cross-cultural validity of the Warwick-Edinburgh Mental Wellbeing Scale in three European populations
Source: BMJ Ment Health. 2025 Mar 11;28(1):e301433. doi: 10.1136/bmjment-2024-301433 (PMC11904331; doi:10.1136/bmjment-2024-301433)
Supplement: online supplemental file 1 [file bmjment-28-1-s001.docx]

**APPENDIX A**

Sociodemographic characteristics of sample by country.

| **Country** | **N** | **N (%) by sex** | | **N (%) of age groups** | | | | | |
| --- | --- | --- | --- | --- | --- | --- | --- | --- | --- |
|  |  | **Male** | **Female** | **16 to 25** | **26 to 34** | **35 to 44** | **45 to 54** | **55 to 64** | **65 years**  **or older** |
| Catalonia (Spain) | 3,651 | 1,812  (49.6) | 1,839  (50.4) | 406  (11.4) | 516  (14.3) | 720  (21.1) | 624  (18.1) | 562  (14.4) | 823  (20.8) |
| Denmark | 3,334 | 1,584  (46.1) | 1,750  (53.9) | 308  (16.0) | 271  (13.2) | 436  (15.8) | 630  (17.2) | 742  (15.0) | 947  (22.8) |
| United Kingdom | 6,955 | 3,052  (49.0) | 3,903  (51.0) | 622  (13.7) | 1,063  (17.0) | 1,081  (16.1) | 1,232  (17.7) | 1,166  (14.3) | 1,791  (21.2) |
| **Total** | **13,940** | **6,448**  **(48.4)** | **7,492**  **(51.6)** | **1,336**  **(13.7)** | **1,850**  **(15.4)** | **2,237**  **(17.3)** | **2,486**  **(17.7)** | **2,470**  **(14.5)** | **3,561**  **(21.5)** |

**APPENDIX B**

Mean score (CI 95%) of the WEMWBS scale by country.


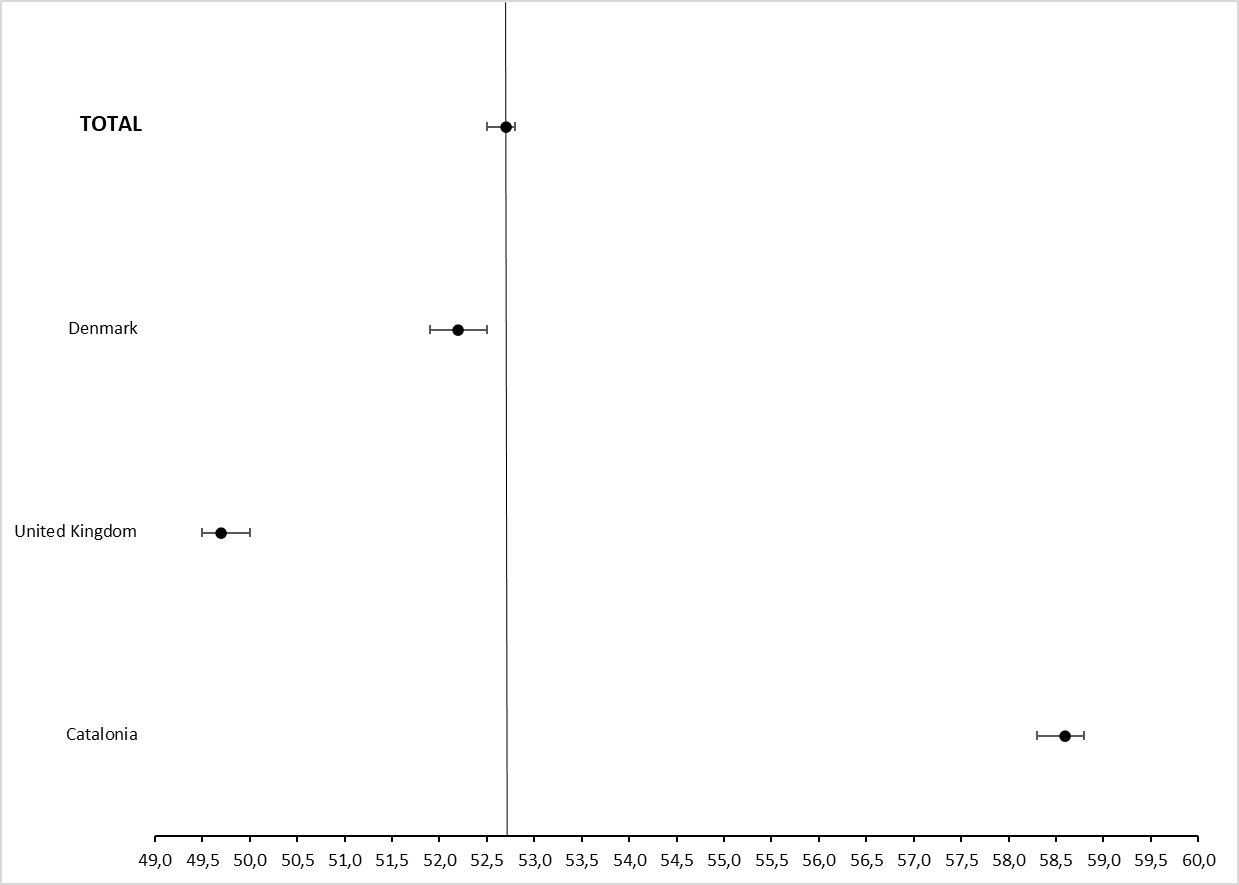


Note: CI: Confidence Interval. The vertical line indicates the total average score of the WEMWBS scale of all countries.

**APPENDIX C**

Fit statistics for different factor structures evaluated on the overall sample and each of the three population groups

| **Population** | ***ꭓ*^2^ (DF)** | **CFI** | **TLI** | **RMSEA (90% CI)** | **SRMR** |
| --- | --- | --- | --- | --- | --- |
| **Catalonia** |  |  |  |  |  |
| M1 – One factor iCFA | 3586,623 (77) | 0.969 | 0.963 | 0.112 (0.109-0.115) | 0.037 |
| M2- 2 Factor iCFA^1^ | 3580.883 (76) | 0.969 | 0.962 | 0.112 (0.109-0.116) | 0.037 |
| M3- 3 Factor iCFA^2^ | 2485,0.21 (74) | 0.978 | 0.973 | 0.094 (0.091-0.098) | 0.030 |
| M4- 3 Factor iCFA^3^ | 2864.753 (74) | 0.975 | 0.969 | 0.102 (0.098-0.105) | 0.033 |
| M5- Second order factor with 3 first order factors ^3^ | 2485.0.21 (74) | 0.978 | 0.973 | 0.094 (0.091-0.098) | 0.030 |
| M6- Bifactor ESEM with target rotation^4^ | 701.972 (41) | 0.994 | 0.987 | 0.066 (0.062-0.071) | 0.013 |
| **Denmark** |  |  |  |  |  |
| M1 – One factor iCFA | 2527.115 (77) | 0.941 | 0.930 | 0.098 (0.094-0.101) | 0.042 |
| M2- 2 Factor iCFA^1^ | 2501.715 (76) | 0.941 | 0.930 | 0.098 (0.095-0.101) | 0.042 |
| M3- 3 Factor iCFA ^2^ | 1504.951 (74) | 0.965 | 0.957 | 0.076 (0.073-0.080) | 0.033 |
| M4- 3 Factor iCFA^3^ | 1976.464 (74) | 0.954 | 0.943 | 0.088 (0.084-0.091) | 0.037 |
| M5- Second order factor with 3 first order factors ^3^ | 1504.951 (74) | 0.965 | 0.957 | 0.076 (0.073-0.080) | 0.033 |
| M6- Bifactor ESEM with target rotation^4^ | 738.611 (41) | 0.983 | 0.963 | 0.071 (0.067-0.076) | 0.020 |
| **United Kingdom** |  |  |  |  |  |
| M1 – One factor iCFA | 7773.787 (77) | 0.943 | 0.933 | 0.120 (0.118-0.022) | 0.043 |
| M2- 2 Factor iCFA^1^ | 7671.924 (76) | 0.944 | 0.933 | 0.120 (0.118-0.122) | 0.043 |
| M3- 3 Factor iCFA ^2^ | 4224.942 (74) | 0.969 | 0.962 | 0.090 (0.088-0.092) | 0.033 |
| M4- 3 Factor iCFA^3^ | 6037.836 (74) | 0.956 | 0.946 | 0.108 (0.105-0.110) | 0.039 |
| M5- Second order factor with 3 first order factors ^3^ | 4224.942 (74) | 0.969 | 0.962 | 0.090 (0.088-0.092) | 0.033 |
| M6- Bifactor ESEM with target rotation^4^ | 1755.195 (41) | 0.987 | 0.972 | 0.078 (0.074-0.081) | 0.017 |
| **Total Sample** |  |  |  |  |  |
| M1 – One factor iCFA | 13116.950 (77) | 0.958 | 0.950 | 0.110 (0.109-0.112) | 0.035 |
| M2- 2 Factor iCFA^1^ | 12956.970 (76) | 0.958 | 0.950 | 0.110 (0.109-0.112) | 0.035 |
| M3- 3 Factor iCFA ^2^ | 7229.000 (74) | 0.977 | 0.972 | 0.083 (0.082-0.085) | 0.027 |
| M4- 3 Factor iCFA ^3^ | 9796.922 (74) | 0.969 | 0.961 | 0.097 (0.095-0.099) | 0.031 |
| M5- Second order factor with 3 first order factors ^2^ | 7220.112 (74) | 0.977 | 0.972 | 0.083 (0.082-0.085) | 0.027 |
| M6- Bifactor ESEM with target rotation^4^ | 3276.205 (41) | 0.990 | 0.977 | 0.075 (0.073-0.077) | 0.014 |

Note*: ꭓ*^2^: Chi-Square test; CFI: Comparative Fit Index; TLI: Tylor-Lewis Index; RMSEA: Root Mean Square Error of Approximation; SRMR: Standardized Root Mean Square Residual; CI: Confidence Interval; ESEM: Exploratory Structural Equation Model; iCFA: categorical item Conformatory Factor Analysis. iCFA model for all countries.^1^. Two factor structure (i.e., items 1,2,3,5,8,12,13 loading on specific factor 1 (hedonia), items 4,6,7,9,11,14 loading on Specific factor 2 (eudaimonia)); ^2^. Three factor structure (i.e., items 1,2,3,5,8,10,13,14 loading on specific factor 1 (hedonia), items 6,7,11 loading on Specific factor 2 (eudaimonia) and items 4,9,12 loading on specific factor 3 (social relationships)); ^3^. Three factor structure (i.e., items 1,3,5,8,10,14 loading on specific factor 1 (hedonia), items 6,7,1,131 loading on Specific factor 2 (eudaimonia) and items 2,4,9,12 loading on specific factor 3 (social relationships)); ^4^. Target rotation with prior specification of the relationship between items and factors (i.e., items 1,2,3,5,8,10,13,14 loading on specific factor 1 (hedonia), items 6,7,11 loading on Specific factor 2 (eudaimonia) and items 4,9,12 loading on specific factor 3 (social relationships)).

**APPENDIX D**

Standardized factor loadings (λ _i_) for general and specific factors of the bifactor ESEM model with target rotation*.

| **Population** | **λ_1_** | **λ_2_** | **λ_3_** | **λ_4_** | **λ_5_** | **λ_6_** | **λ_7_** | **λ_8_** | **λ_9_** | **λ_10_** | **λ_11_** | **λ_12_** | **λ_13_** | **λ_14_** |
| --- | --- | --- | --- | --- | --- | --- | --- | --- | --- | --- | --- | --- | --- | --- |
| **Catalonia** |  |  |  |  |  |  |  |  |  |  |  |  |  |  |
| General Factor | **0.54** | **0.80** | **0.52** | **0.73** | **0.62** | **0.87** | **0.89** | **0.88** | **0.86** | **0.91** | **0.90** | **0.83** | **0.61** | **0.76** |
| Specific Factor 1 | 0.40 |  | 0.34 |  | 0.55 |  |  |  |  |  |  |  | 0.35 | 0.41 |
| Specific Factor 2 |  |  |  |  | 0.22 | 0.26 | 0.21 |  |  |  |  |  |  |  |
| Specific Factor 3 |  |  |  | 0.55 |  |  |  |  | 0.21 |  |  |  |  |  |
| **Denmark** |  |  |  |  |  |  |  |  |  |  |  |  |  |  |
| General Factor | **0.77** | **0.72** | **0.71** | **0.59** | **0.78** | **0.76** | **0.71** | **0.83** | **0.69** | **0.79** | **0.60** | **0.60** | **0.68** | **0.86** |
| Specific Factor 1 | 0.22 | 0.22 |  |  |  |  |  |  |  |  |  |  | 0.34 |  |
| Specific Factor 2 |  |  |  |  |  | 0.26 | 0.38 |  |  | 0.26 | 0.43 |  |  |  |
| Specific Factor 3 |  |  |  | 0.29 |  |  |  |  | 0.73 |  |  | 0.32 |  |  |
| **United Kingdom** |  |  |  |  |  |  |  |  |  |  |  |  |  |  |
| General Factor | **0.59** | **0.69** | **0.73** | **0.56** | **0.65** | **0.76** | **0.77** | **0.89** | **0.61** | **0.87** | **0.70** | **0.63** | **0.71** | **0.86** |
| Specific Factor 1 | 0.37 | 0.32 |  | 0.23 |  |  |  |  |  |  |  |  |  |  |
| Specific Factor 2 |  |  |  |  |  | 0.34 | 0.46 |  |  |  | 0.32 |  |  |  |
| Specific Factor 3 |  |  |  | 0.32 |  |  |  |  | 0.63 |  |  | 0.40 |  |  |
| **Total** |  |  |  |  |  |  |  |  |  |  |  |  |  |  |
| General Factor | **0.63** | **0.75** | **0.70** | **0.64** | **0.73** | **0.81** | **0.80** | **0.89** | **0.73** | **0.88** | **0.74** | **0.69** | **0.69** | **0.87** |
| Specific Factor 1 | 0.34 | 0.28 |  | 0.23 | 0.23 |  |  |  |  |  |  |  |  |  |
| Specific Factor 2 |  |  |  |  |  | 0.30 | 0.39 |  |  |  | 0.28 |  |  |  |
| Specific Factor 3 |  |  |  | 0.36 |  |  |  |  | 0.52 |  |  | 0.34 |  |  |

λi: Standardized factor loading for the specific item (i). Only factor loadings with absolute value >=0.2 are presented. *Target rotation with prior specification of the relationship between items and factors following the three-factor structure by Sarasjärvi et al 2023 (i.e., items 1,2,3,5,8,10,13,14 loading on specific factor 1 (hedonia), items 6,7,11 loading on Specific factor 2 (eudaimonia) and items 4,9,12 loading on specific factor 3 (social relationships)).

**APPENDIX E**

Psychometric indices for the interpretation of the bifactor ESEM model, for each population group and the total sample

|  | **Catalonia** | **Denmark** | **United Kingdom** | **Total** |
| --- | --- | --- | --- | --- |
| **Omega** | 0.97 | 0.95 | 0.95 | 0.96 |
| **Average Relative Bias** | 0.04 | 0.04 | 0.04 | 0.03 |
| **Omega H** |  |  |  |  |
| General factor | 0.92 | 0.92 | 0.92 | 0.93 |
| Specific factor 1 | 0.04 | 0.00 | 0.00 | 0.01 |
| Specific factor 2 | 0.02 | 0.01 | 0.01 | 0.01 |
| Specific factor 3 | 0.04 | 0.02 | 0.02 | 0.01 |
| **ECV** |  |  |  |  |
| General factor | 0.85 | 0.82 | 0.82 | 0.86 |
| Specific factor 1 | 0.08 | 0.04 | 0.04 | 0.04 |
| Specific factor 2 | 0.03 | 0.06 | 0.06 | 0.04 |
| Specific factor 3 | 0.04 | 0.08 | 0.08 | 0.06 |

ESEM: Exploratory Structural Equation Model; ECV: Explained Common Variance.

**APPENDIX F**

Akaike Information Criterion (AIC) for the different adjusted weighted polytomous IRT models.

| **Country** | **N** | **PCM** | **GPCM** | **GRM** |
| --- | --- | --- | --- | --- |
| Catalonia | 3,651 | 9.02 | 8.63 | 8.45 |
| Denmark | 3,334 | 9.60 | 9.51 | 9.45 |
| United Kingdom | 6,955 | 20.74 | 20.33 | 20.06 |
| **Total** | **13,940** | 40.08 | 39.38 | 38.78 |

Note: The table shows the AIC x 10 ^4^;.PCM: Partial Credit Model; GPCM: Generalized Partial Credit Model; GRM: Graded

**APPENDIX G**

Discrimination capacity of the items (α _i_) from Graded Response Model (GRM).

| **Country** | **N** | **α_1_** | **α_2_** | **α_3_** | **α_4_** | **α_5_** | **α_6_** | **α_7_** | **α_8_** | **α_9_** | **α_10_** | **α_11_** | **α_12_** | **α_13_** | **α_14_** |
| --- | --- | --- | --- | --- | --- | --- | --- | --- | --- | --- | --- | --- | --- | --- | --- |
| Catalonia | 3,651 | 1.29 | 2.61 | 1.24 | 2.20 | 1.67 | 3.77 | 3.87 | 3.72 | 3.32 | 4.17 | 3.67 | 2.74 | 1.57 | 2.41 |
| Denmark | 3,334 | 2.13 | 1.98 | 1.69 | 1.41 | 2.10 | 2.38 | 2.02 | 2.86 | 1.96 | 2.57 | 1.49 | 1.41 | 1.66 | 2.68 |
| United Kingdom | 6,955 | 1.42 | 1.93 | 1.93 | 1.30 | 1.45 | 2.48 | 2.61 | 3.55 | 1.52 | 3.43 | 2.01 | 1.48 | 1.74 | 2.95 |
| **Total** | **13,940** | **1.56** | **2.34** | **1.72** | **1.72** | **1.91** | **2.99** | **2.83** | **3.72** | **2.15** | **3.65** | **2.26** | **1.75** | **1.79** | **2.89** |

Note: the higher the discrimination parameter, the greater the ability of that item to discriminate whether a person presents with features of mental well-being. α i: Discrimination capacity of the specific item (i).

**APPENDIX H**

Total Test Information Function (TIF) overall and by country.

Note: The dashed line at *Information* = 10 indicates a reliability of 0.90; dashed line at *Information* = 5 indicates a reliability of 0.80
